# RETRACTION: Lnc‐GIHCG Promotes Cell Proliferation and Migration in Gastric Cancer through miR‐ 1281 Adsorption

**DOI:** 10.1002/mgg3.70271

**Published:** 2026-07-14

## Abstract

**RETRACTION**: LiuG. , JiangZ. , QiaoM. , and WangF. , “Lnc‐GIHCG Promotes Cell Proliferation and Migration in Gastric Cancer through miR‐ 1281 Adsorption,” Molecular Genetics & Genomic Medicine 7, no. 6 (2019): e711, 10.1002/mgg3.711.31050210 PMC6565586

The above article, published online on 02 May 2019 in Wiley Online Library (onlinelibrary.wiley.com), has been retracted by agreement between the journal Editor‐in‐Chief, Paraminder Dhillon; and Wiley Periodicals LLC.

Following publication, a third party raised concerns regarding similarities between the SGC‐7901 panels of Figure 5e (TLE1 & TLE1+miR‐1281), Figure 2c and Figure 3h (miR‐1281 mimics); with images published by different authors in a different scientific context [1]. Further investigation by the publisher found overlap between Figure 2c (panel HGC‐27/GIHCG) and Figure 5e (panel HGC‐27/TLE1) within this article. The authors did not respond to requests for an explanation or for the original data. The retraction has been agreed because the identified flaws affect the interpretation of the data and results presented.

The authors were informed of the decision to retract.

References

[1] LiH. , YuB. , LiJ. , SuL. , YanM. , ZhuZ. , and LiuB. , “Overexpression of lncRNA H19 Enhances Carcinogenesis and Metastasis of Gastric Cancer,” Oncotarget 5, no. 8 (2014): 2318–2329, 10.18632/oncotarget.1913.24810858 PMC4039165

**RETRACTION**: G. Liu , Z. Jiang , M. Qiao , and F. Wang , “Lnc‐GIHCG Promotes Cell Proliferation and Migration in Gastric Cancer through miR‐ 1281 Adsorption,” Molecular Genetics & Genomic Medicine 7, no. 6 (2019): e711, 10.1002/mgg3.711.31050210 PMC6565586

The above article, published online on 02 May 2019 in Wiley Online Library (onlinelibrary.wiley.com), has been retracted by agreement between the journal Editor‐in‐Chief, Paraminder Dhillon; and Wiley Periodicals LLC.

Following publication, a third party raised concerns regarding similarities between the SGC‐7901 panels of Figure 5e (TLE1 & TLE1+miR‐1281), Figure 2c and Figure 3h (miR‐1281 mimics); with images published by different authors in a different scientific context [1]. Further investigation by the publisher found overlap between Figure 2c (panel HGC‐27/GIHCG) and Figure 5e (panel HGC‐27/TLE1) within this article. The authors did not respond to requests for an explanation or for the original data. The retraction has been agreed because the identified flaws affect the interpretation of the data and results presented.

The authors were informed of the decision to retract.

References

[1] H. Li , B. Yu , J. Li , L. Su , M. Yan , Z. Zhu , and B. Liu , “Overexpression of lncRNA H19 Enhances Carcinogenesis and Metastasis of Gastric Cancer,” Oncotarget 5, no. 8 (2014): 2318–2329, 10.18632/oncotarget.1913.24810858 PMC4039165

